# Supplementary material for: Digital Online Patient Informed Consent for Anesthesia before Elective Surgery—Recent Practice in Europe
Source: Healthcare (Basel). 2023 Jul 5;11(13):1942. doi: 10.3390/healthcare11131942 (PMC10340391; doi:10.3390/healthcare11131942)
Supplement: Supplementary file 1 [file healthcare-11-01942-s001.zip › healthcare-2427970-supplementary.pdf]

Table S1: Gross Domestic Product per capita (in billions US$) for the year 2021

Country	GDPPC per capita (billions)	Group
Liechtenstein	169,049.2	High
Luxembourg	135,682.8	High
Switzerland	93,457.4	High
Ireland	99,152.1	High
Norway	89,202.8	High
Denmark	67,803.0	High
Iceland	68,383.8	High
Netherlands	58,061.0	High
Sweden	60,239.0	High
Finland	53,982.6	High
Austria	53,267.9	High
Germany	50,801.8	High
Belgium	51,767.8	High
Israel	51,430.1	High
United Kingdom (UK)	47,334.4	High
France	43,518.5	Middle
Italy	35,551.3	Middle
Malta	33,257.4	Middle
Cyprus	30,798.5	Middle
Spain	30,115.7	Middle
Slovenia	29,200.8	Middle
Estonia	27,280.7	Middle
Czech Republic	26,378.5	Middle
Portugal	24,262.2	Middle
Lithuania	23,433.4	Middle
Slovak Republic	21,087.8	Middle
Latvia	20,642.2	Middle
Greece	20,276.5	Middle
Hungary	18,772.7	Middle
Poland	17,840.9	Middle
Croatia	17,398.8	Low
Romania	14,861.9	Low

Russian Federation	12,172.8	Low
Bulgaria	11,635.0	Low
Kazakhstan	10,041.5	Low
Turkiye	9,586.6	Low
Serbia	9,215.0	Low
Montenegro	9,367.0	Low
Belarus	7,303.7	Low
Bosnia and Herzegovina	6,916.4	Low
North Macedonia	6,720.9	Low
Albania	6,494.4	Low
Moldova	5,314.5	Low
Kosovo	4,986.6	Low
Ukraine	4,835.6	Low
Uzbekistan	1,983.1	Low
